# Genome-wide identification and expression analysis of *C3H* gene family in melon

**DOI:** 10.3389/fpls.2025.1500429

**Published:** 2025-03-13

**Authors:** Ling Zheng, Haifang Dai, Yuanfang Mu, Jinbo Li, Yanwei Cheng, Jianming Han

**Affiliations:** ^1^ Department of Biology, Luoyang Normal University, Henan, Luoyang, China; ^2^ School of Biological Sciences, Henan University of Science and Technology, Henan, Xinxiang, China

**Keywords:** *Cucumis melo*, C3H gene family, drought stress, heavy metal lead stress, fusarium wilt infection

## Abstract

Zinc finger protein (ZFP) represent a significant class of transcription factors in plants, involved in various functions, including tissue development, signal transduction, and responses to both biotic and abiotic stresses. ZFPs are categorized into 10 distinct subfamilies, among which the C3H gene family is recognized as a functionally significant group of transcription factors.To date, no studies have been reported regarding the *C3H* gene family in melon (*Cucumis melo*). In this study, 38 *CmC3H* genes were identified in the melon genome, and these genes are unevenly distributed across the 12 chromosomes. Phylogenetic analysis classified the *C3H* family members into four groups, with significant differences observed in sequence, protein motifs, and gene structure among *CmC3H* genes within the same group. The *CmC3H* family contains one pair of segmentally duplicated genes and shares 20, 7, 39, and 38 pairs of homologous *C3H* genes with *Arabidopsis thaliana*, rice (*Oryza sativa*), cucumber (*Cucumis sativus*), and watermelon (*Citrullus lanatus*), respectively. Promoter region analysis revealed a high abundance of *cis*-elements associated with growth and development, hormone regulation, and stress responses. Expression profiling revealed that *CmC3H* family members exhibit significant tissue-specific expression patterns. Quantitative PCR analysis indicated that six genes (*CmC3H4*, *CmC3H7*, *CmC3H13*, *CmC3H24*, *CmC3H33*, and *CmC3H38*) may play roles in melon’s drought stress resistance. Heavy metal lead stress appears to suppress the expression of *CmC3H* genes. The genes *CmC3H24* and *CmC3H33* may be involved in regulating melon’s resistance to *Fusarium wilt* infection. *CmC3H11* and *CmC3H21* can be considered as the key candidate genes for improving the melon’s ability to resist both biotic and abiotic stresses.This study provides preliminary insights into the expression profiles of *CmC3H* genes under drought stress, heavy metal lead stress, and *Fusarium wilt* infection, offering a theoretical foundation for the molecular mechanisms underlying melon improvement and stress resistance.

## 1 Introduction

Zinc finger proteins (ZFPs) represent one of the largest and most specific families of transcription factors in plants. ZFPs are classified into 10 major types based on the number of cysteine and histidine residues and the spacing of amino acids between them: C2H2, C2HC, C2HC5, C2C2, C3H, C3HC4, C4, C4HC3, C6, and C8 (Kamaliyan and Clarke, 2024). The *C3H* gene family, a significant subgroup of this group, is characterized by the presence of one or more C3H-type zinc finger domains, each consisting of three cysteine and one histidine residue bound to a zinc ion. This characteristic C3H-type zinc finger motif is commonly found in eukaryotes and is named for its distinctive structure (Bogamuwa and Jang, 2014; Liu et al., 2022). Initially, the consensus sequence of the C3H motif was defined as C-X6-14-C-X4-5-C-X3-H (where X represents any amino acid), based on variations in the spacing of amino acids between cysteine and histidine in the motif. Subsequent studies have redefined the C3H motif as C-X4-17-C-X4-6-C-X3-H (Berg and Shi, 1996; Rameneni et al., 2018). *C3H* genes play crucial roles in both plants and animals by specifically binding to RNA or DNA, participating in various biological processes, including the regulation of gene expression, RNA processing, disease response, and adaptation to environmental stresses (Cheng et al., 2020; Velásquez et al., 2024). Research has demonstrated that members of the *C3H* gene family are essential for processes such as photoperiod regulation, hormone signaling, and disease resistance in plants, particularly in modulating responses to stresses like drought, salinity, and pathogen infection (Han et al., 2021). Advancements in genomics and functional genomics technologies have facilitated the identification of additional *C3H* gene family members, providing new insights into their roles in plant physiology and molecular mechanisms (Ai et al., 2022).

To date, the *C3H* gene family has been studied in several plants, including *A. thaliana* (Wang et al., 2008), rice (Wang et al., 2008), maize (Peng et al., 2012), alfalfa (Zhang et al., 2013), citrus (Liu et al., 2014), and poplar (Chai et al., 2012), with the functions of several *C3H* genes validated across species. In *A. thaliana*, the overexpression of *AtC3H17*, *AtC3H29*, and *AtC3H47* has been shown to enhance salt tolerance (Seok et al., 2018; Sun et al., 2007), whereas the overexpression of *AtC3H49* improves tolerance to drought as well as salt stress (Lee et al., 2012). Overexpression of *GhZFP1* in transgenic tobacco significantly enhances salt tolerance by influencing Na^+^ homeostasis and K^+^ acquisition (Guo et al., 2009). The overexpression of *BoC3H* improves salt tolerance in transgenic cabbage (Jiang et al., 2017), while *PeC3H74* transgenic Arabidopsis plants exhibit stronger drought tolerance compared to wild-type plants (Chen et al., 2020a). Regarding biotic stress in plants, the nuclear protein *GHZFP1* in cotton interacts with *GZIRD21A* and *GZIPR5*, contributing to enhanced fungal disease resistance (Guo et al., 2009). In chili pepper, the *C3H* gene *CaC3H1* modulates the antagonistic interaction between salicylic acid (SA) and jasmonic acid (JA)/ethylene (ET) signaling pathways, thus enhancing resistance to bacterial wilt caused by *Ralstonia solanacearum* (Lei, 2014). In *A. thaliana*, the *C3H15* gene has been found to negatively regulate cell elongation by inhibiting brassinosteroid signaling (Chai et al., 2022). In rice, the C3H-type genes *OsLIC* and *OsDOS* are involved in the regulation of brassinosteroid signaling, affecting structural development and delaying leaf senescence, respectively (Wang et al., 2008; Kong et al., 2006). *ZmC3H9* (maize) is implicated in the regulation of phenolic compound biosynthesis (Abnave et al., 2024). The *GhTZF1* gene regulates leaf senescence in cotton (Zhou et al., 2014). *PEI1* plays a crucial role during Arabidopsis embryogenesis and is an embryo-specific *C3H* zinc finger gene that primarily functions in the apical domain of the embryo (Li and Thomas, 1998). The *C3H* gene *AtC3H14* (*At1g66810*) has been identified as a key regulator of secondary cell wall biosynthesis in *A. thaliana* (Ko et al., 2009).

Melon (*C. melo* L.) is a member of the Cucurbitaceae family and is valued for its juicy fruit, rich in carbohydrates and nutrients, making it economically important. However, melon cultivation is often hindered by various stress factors that reduce fruit yield and quality. In Henan Province, China, melon is widely cultivated, but its production is frequently threatened by *Fusarium wilt*, which can cause plant mortality. Moreover, Henan Luoyang, situated in the middle reaches of the Yellow River, experiences a semi-arid to semi-humid climate(Shen et al., 2023). Melon cultivation in this region is highly susceptible to drought stress. Industrial development has also led to heavy metal pollution in some soils, further compromising melon growth and development. Despite these challenges, no research has been conducted on the *C3H* gene family in melon. Melon, the second Cucurbitaceae crop to have its genome sequenced, has a chromosome number of 2n = 2x = 24 and an approximate genome size of 450 Mb (Garcia-Mas et al., 2012). The updated, high-quality melon genome sequence provides an opportunity for the identification and characterization of the *CmC3H* family. Thus, this study aims to identify members of the *C3H* gene family in melon and perform bioinformatic analyses, including assessments of protein physicochemical properties, phylogeny, conserved motifs, gene structure, gene duplication, *cis*-elements, and expression patterns. Additionally, the study investigates the expression of 10 selected *CmC3H* genes in the ‘Super Sweet White Sugar’ melon variety under drought stress, lead (Pb) heavy metal stress, and *Fusarium wilt* infection using quantitative PCR analysis. This research provides valuable insights into the structure and function of *CmC3H* genes.

## 2 Materials and methods

### 2.1 Identification, localization, and phylogenetic tree construction of melon C3H family members

The melon genome sequence DHL92 v4.0 was downloaded from the CuGenDB database (http://cucurbitgenomics.org/) to identify *C3H* genes (Garcia-Mas et al., 2012). Using the Hidden Markov Model (HMM) profile PF00642 (https://www.ebi.ac.uk/interpro/entry/pfam/#table), a genome-wide search was conducted on the melon genome protein data using the HMMER tool, retaining genes with an e-value of less than or equal to e^−5^ (Potter et al., 2018). Additionally, 50 Arabidopsis C3H protein sequences were downloaded from the UniProt database (https://www.uniprot.org/) (UniProt Consortium, 2021) and subjected to a BLAST search against the Arabidopsis C3H protein sequences with an e-value of less than or equal to e^−5^ (Ye et al., 2006). After removing redundant sequences, the remaining dataset could be used for further analysis. Ultimately, 38 *C3H* genes were identified in the melon genome, and their chromosomal distribution was mapped using the TBtools (V2.131) (Chen et al., 2020b). Following the same methodology, 35 and 38 *C3H* members were identified in cucumber and watermelon, respectively. Protein physicochemical properties were analyzed using ExPASy (http://web.expasy.org/protparam/) (Wilkins et al., 1999), and subcellular localization was predicted using WoLF PSORT II (https://www.genscript.com/wolf-psort.html?src=leftbar) (Horton et al., 2007). Finally, a phylogenetic tree of the melon *C3H* gene family was constructed following a previous study (Deng et al., 2023). This process involved using MEGA 11 software to perform a ClustalW multiple sequence alignment of the *CmC3H* and *AtC3H* sequences and constructing the phylogenetic tree with the Neighbor-Joining (NJ) method, with 1,000 bootstrap replications, using the Poisson correction model and the pairwise deletion option (Deng et al., 2023).

### 2.2 Conserved motif, domain, and gene structure

Conserved motifs were identified in the 38 CmC3H protein sequences using the MEME tool (http://meme-suite.org/tools/meme), specifying a maximum of 10 motifs with default parameters (Bailey et al., 2009). The positions and numbers of C3H domains within the CmC3H protein sequences were analyzed using the NCBI CDD online tool (https://www.ncbi.nlm.nih.gov/cdd/) (Marchler-Bauer et al., 2015). Exon and intron positions and numbers within the *CmC3H* genes were identified based on melon genome data. The phylogenetic tree, conserved motifs, domains, and gene structures of *CmC3H* family members were subsequently clustered and visualized through TBtools (V2.131).

### 2.3 Gene duplication and Ka/Ks analysis

Gene duplication, including segmental and tandem duplications, among the 38 *CmC3H* members was analyzed using the MCScanX tool (Wang et al., 2012). Constructing chromosome homology maps using SRplot online (https://www.bioinformatics.com.cn/?keywords=circos) Rcircos circle diagram tool (Tang et al., 2023a). Homologous C3H gene pairs between melon and Arabidopsis, rice, cucumber, and watermelon were identified. The Ka (nonsynonymous substitution rate), Ks (synonymous substitution rate), and Ka/Ks ratios of all duplicated genes were computed through the KaKs_Calculator tool(Version 3.0) (Zhang et al., 2006).

### 2.4 Cis-element annotation

Promoter sequences (2,000 bp upstream) of the 38 *CmC3H* genes were extracted and annotated to identify *cis*-elements through the PlantCARE online tool (http://bioinformatics.psb.ugent.be/webtools/plantcare/html/) (Lescot et al., 2002). The positional distribution of *cis*-elements related to abiotic and biotic stresses, phytohormone responsiveness, and plant growth and development was mapped according to previous studies (Zhang et al., 2024).

### 2.5 Expression pattern analysis

The expression patterns of the *CmC3H* family members were analyzed by retrieving FPKM (Fragments Per Kilobase of exon model per Million mapped fragments) values from the melon transcriptome database (https://melonet-db.dna.affrc.go.jp/ap/top) across eleven distinct tissues: dry seeds, root, middle stem, upper stem, leaves, tendril, petal, stigma, ovary at 4 Days After Flowering (DAF4), fruit flesh at 50 Days After Flowering (DAF50), and fruit epicarp at 50 Days After Flowering (DAF50) (Yu et al., 2023). The expression pattern of the *CmC3H* family was visualized using TBtools (V2.131). In this figure, the expression levels are represented by color intensity, with darker colors corresponding to higher expression levels.

### 2.6 Target gene and protein interaction prediction

The protein sequences of *CmC3H* family members were uploaded to the STRING database (https://string-db.org/) for node comparison, with interactions among CmC3H members predicted based on Arabidopsis protein interaction data (Szklarczyk et al., 2023). The binding motif profile for the Arabidopsis C3H12 transcription factor (MA1756.2) was obtained from the JASPAR Plantae database (https://jaspar.elixir.no/search?q=&collection=CORE&tax_group=plants) (Castro-Mondragon et al., 2022). Subsequently, the 2,000 bp promoter sequences of all genes in the melon genome were extracted and analyzed using the motif FIMO tool (https://meme-suite.org/meme/) to detect genes potentially binding with the *C3H* transcription factor. Finally, target gene domain prediction was performed using the PFAM database (Mistry et al., 2021), and KEGG (Kyoto Encyclopedia of Genes and Genomes) and GO (Gene Ontology) enrichment analyses were conducted on the target genes using OmicShare Tools (https://www.omicshare.com/tools).

### 2.7 Quantitative real-time PCR

The seeds of the melon variety ‘Super Sweet White Sugar Jar’ were soaked, germinated, and then sown. The plants were grown in a greenhouse under a 16-hour light/8-hour dark photoperiod, with temperatures maintained at 28°C during the day and 18°C at night, until they reached the three-leaf stage. *Fusarium oxysporum* was isolated from wilted melon plants, and a spore suspension with a concentration of 1×10^8^ spores/mL was prepared. The plants were inoculated via root irrigation, applying 10 mL of spore suspension per plant. Root, middle stem, and first true leaf samples were collected at 0 hours (CK), 12 hours, 24 hours, and 48 hours post-inoculation.

Drought stress treatment was applied by placing the three-leaf stage melon plants in a 15% PEG6000 solution, and leaf samples were collected at 0 hours (CK), 12 hours, 24 hours, and 48 hours. For heavy metal lead stress treatment, Pb (NO_3_)_2_ was dissolved in distilled water and applied to the pots, bringing the Pb ion concentration in the soil to 2000 mg/kg. Leaf samples were collected at 0 hours (CK), 12 hours, 24 hours, and 48 hours. All experiments were conducted with three biological replicates, and the collected samples were immediately placed in liquid nitrogen and stored at -80°C for further analysis.

The expression levels of 10 *CmC3H* genes at four time points (0 hours, 12 hours, 24 hours, and 48 hours) under drought stress (leaves; three biological replicates), heavy metal lead stress (leaves; three biological replicates), and *Fusarium wilt* infection (roots, stems, and leaves; three biological replicates) were analyzed using quantitative real-time PCR (qRT-PCR).The qRT-PCR primers for the selected *CmC3H* genes were designed using Primer Premier 5 (Singh et al., 1998). Total RNA was extracted from all samples using the Spectrum™ Plant Total RNA Kit (Merck KGaA), with the quality and concentration assessed using a Nanodrop 2000. First-strand cDNA was synthesized using the SweScript All-in-One First Strand cDNA Synthesis kit (TRANS, G3337). The qPCR reactions were performed using a CFX96 Real-Time PCR Detection System (Bio-Rad, Hercules, CA) with 2×SYBR Green qPCR Master Mix (no ROX) (TRANS, G3320). The cycling conditions were set as follows: 95°C for 30 seconds of pre-denaturation, followed by 40 cycles of 95°C for 15 seconds and 60°C for 30 seconds. The LOC103490203 gene was used as the internal reference gene in melon, and the relative expression levels of the target genes were calculated using the 2^−ΔΔCT^ method (Livak and Schmittgen, 2001). Differences among treatments were assessed by the least significance difference using one-way analysis in SPSS 19.0 (SPSS China, Beijing, China).

### 2.8 Subcellular localization

The subcellular localization of the target gene was predicted using the WOLF PSORT website. The coding sequence of *CmC3H21* was cloned into the pBI121-GFP vector to construct the pBI121-CmC3H21-GFP recombinant plasmid. Subsequently, the pBI121-CmC3H21-GFP plasmid and mCherry (a nuclear marker) were introduced into *Agrobacterium* tumefaciens strain GV3101. The *Agrobacterium* culture was activated and grown until the OD600 reached 1.5, followed by centrifugation to remove the supernatant. The pellet was resuspended in an infiltration buffer (10 mM MES, 10 mM MgCl_2_, and 200 µM acetosyringone) and vortexed to adjust the OD600 to 1.0. The resulting suspension was infiltrated into the abaxial side of leaves from approximately five-week-old tobacco (*Nicotiana benthamiana*) seedlings. After infiltration, the plants were kept in the dark at 22°C for 48 hours. The infiltrated leaves were then excised and imaged using a confocal laser scanning microscope (TCS SP8, Leica) to visualize the subcellular localization of the fluorescently tagged proteins.

## 3 Results

### 3.1 Distribution and physicochemical properties of CmC3H genes

**Figure 1:**
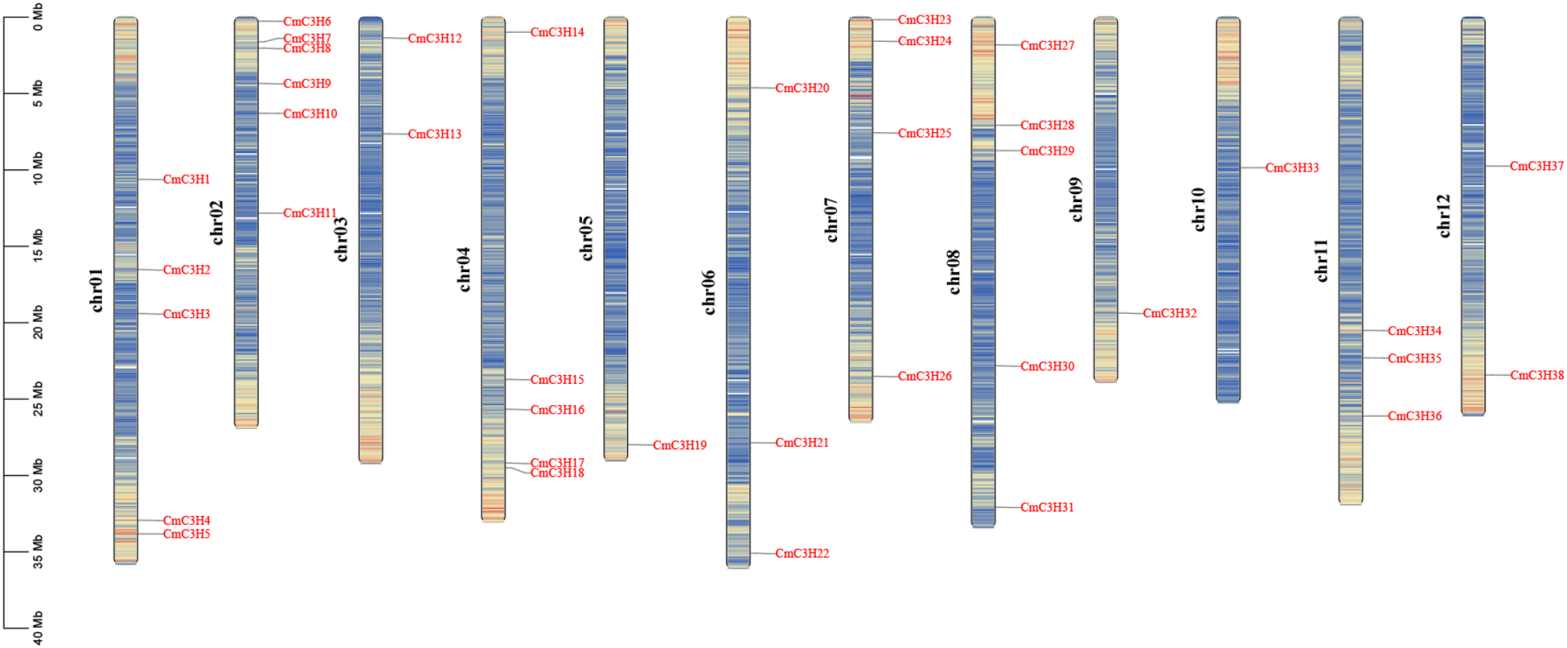
Chromosomal distribution of *CmC3H* family members. The left scale represents the chromosome length, with “chr” denoting the chromosome. Gene density on each chromosome was calculated over a genetic distance of 200 kb and is represented by a gradient color from blue (low gene density) to red (high gene density). White areas indicate regions lacking gene distribution information.

A total of 38 *C3H* genes were identified in the melon genome, and these are unevenly distributed across 12 chromosomes (**Figure 1**). Specifically, the genes are distributed as follows: 5, 6, 2, 5, 1, 3, 4, 5, 1, 1, 3, and 2 on chromosomes Chr01 to Chr12, respectively. These genes were renamed *CmC3H1* through *CmC3H38* based on their chromosomal locations. The physicochemical properties of the *CmC3H* family members were further analyzed (**Supplementary Table S1**) (“aa” denotes amino acids, and “Da” refers to Daltons). The analysis indicated that the number of amino acids in CmC3H members ranges from 202 aa (CmC3H1) to 1034 aa (CmC3H25), with molecular weights varying from 22128.07 Da (CmC3H1) to 114623.34 Da (CmC3H25). The Instability Index ranges from 28.7 (CmC3H16) to 82.26 (CmC3H29), while the Aliphatic Index varies from 36.53 (CmC3H31) to 78.96 (CmC3H16). All CmC3H members are hydrophilic proteins. The theoretical isoelectric points (pI) range from 4.92 to 9.65, with 17 members having a pI below 7 and 21 members having a pI above 7. Subcellular localization analysis revealed that three genes, *CmC3H9*, *CmC3H20*, and *CmC3H31*, lack a start codon, preventing the prediction of their subcellular localization. Among the remaining members, 31 are predicted to localize in the nucleus, 3 in the chloroplast, and 1 in the cytoplasm.

### 3.2 Phylogenetic analysis of the CmC3H gene family

**Figure 2:**
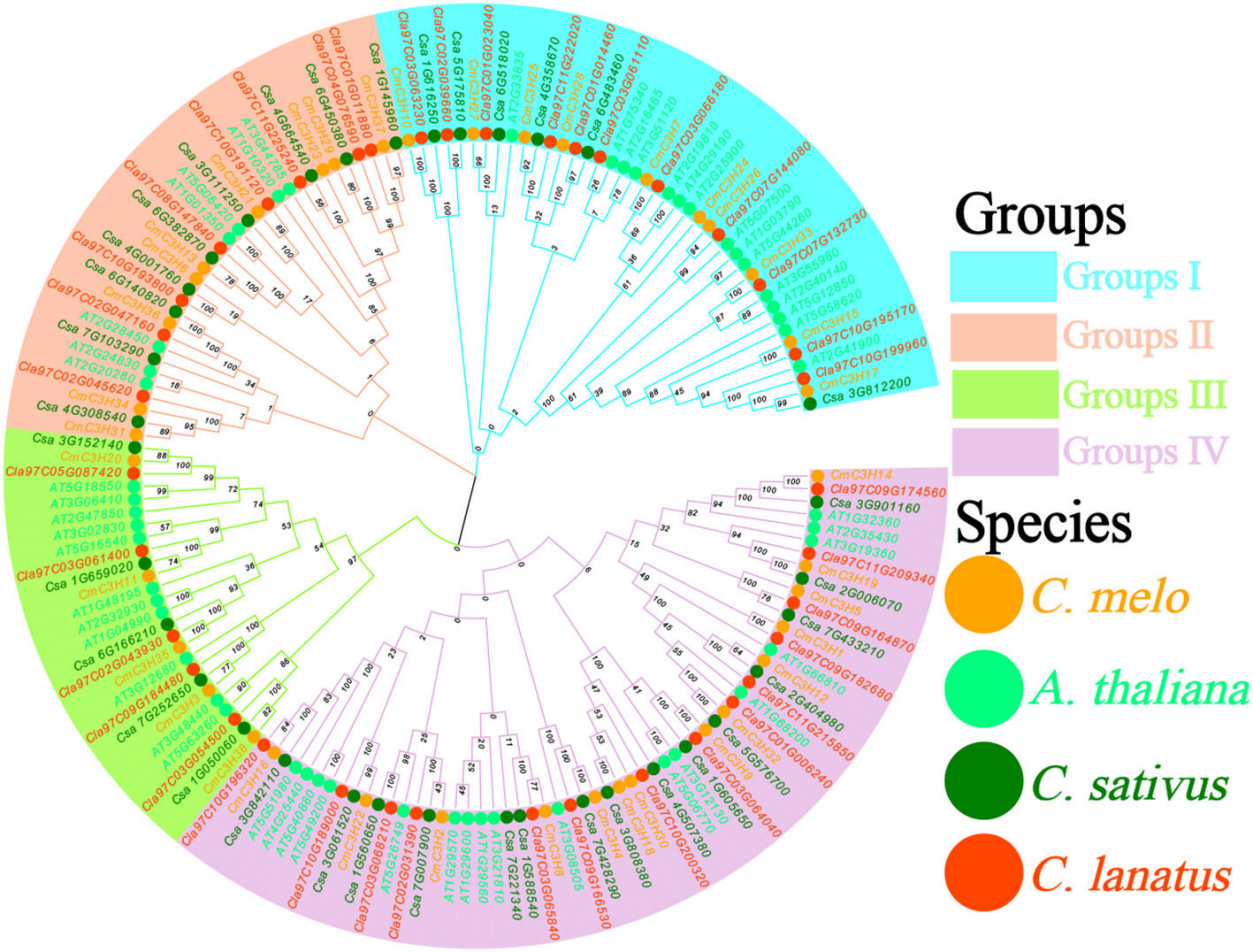
Neighbor-Joining phylogenetic tree of *C3H* family members from *A. thaliana*, *C. melo*, *C. sativus*, and *C. lanatus*. The four differently colored regions correspond to the four groups, and the solid circles of different colors represent the four species.

A Neighbor-Joining phylogenetic tree of *C3H* family members from *A. thaliana*, melon (*C. melo*), cucumber (*C. sativus*), and watermelon (*C. lanatus*) was constructed using MEGA 11. The resulting tree was categorized into four groups: Group I, Group II, Group III, and Group IV (**Figure 2**; **Supplementary Table S2**).Groups I, II, III, and IV contained 42, 33, 26, and 60 members, respectively. The *CmC3H* genes were distributed across these groups, with 10, 9, 5, and 14 genes in Groups I to IV, respectively, accounting for 26.3%, 23.7%, 13.2%, and 36.8% of the total. The *C3H* family consisted of 38 members, while cucumber and watermelon had 35 and 38 *C3H* genes, respectively, indicating similar gene numbers among the three species. Clustering analysis revealed that *C3H* genes in melon, cucumber, and watermelon were highly conserved, suggesting strong evolutionary conservation among these closely related species. Analysis of motifs and domains in the *CmC3H* family revealed that members within the same subgroup shared similar motif types and domain structures. These findings support the phylogenetic subgroup classification based on evolutionary analysis and further validate the rationale behind subgroup categorization in the evolutionary tree (Liu et al., 2024).

### 3.3 Analysis of protein motifs and gene structure of CmC3H family members

**Figure 3:**
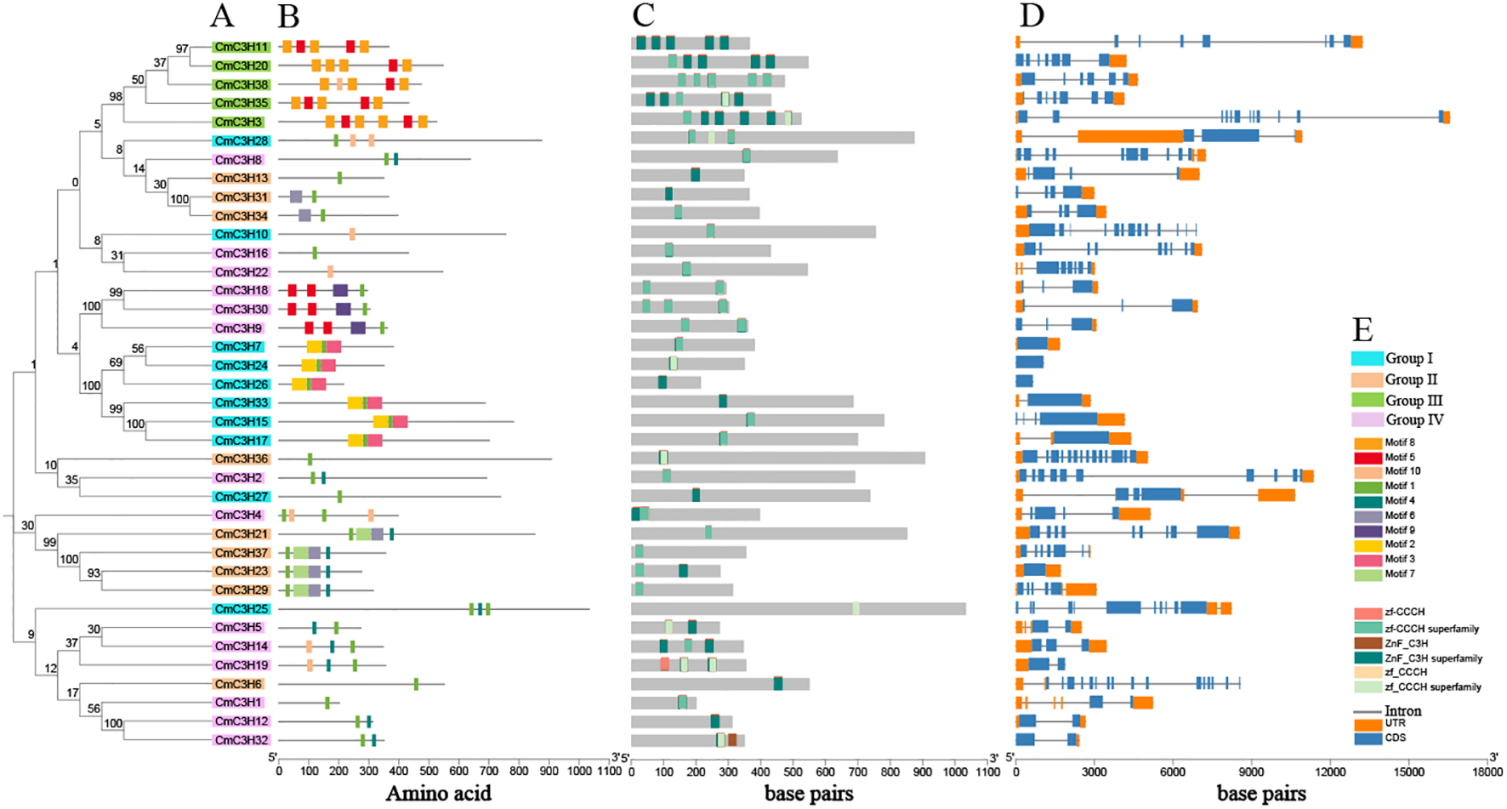
Analysis of the phylogenetic tree, motifs, domains, and gene structure of *CmC3H* family members. **(A)** Neighbor-Joining phylogenetic tree. **(B)** Conserved protein motifs. **(C)** Distribution of GATA domains. **(D)** Exon and intron positions. **(E)** Color-coded rectangles and their corresponding types.

A total of 10 motifs were annotated in the protein sequences of *CmC3H* family members. A Neighbor-Joining phylogenetic tree was further constructed specifically for *CmC3H* family members (**Figure 3A**). The analysis indicated that, unlike the broader phylogenetic distribution shown in **Figure 2**, the *CmC3H* members within the same group were not strictly clustered within the same major branches. Additionally, significant differences in the types and numbers of conserved protein motifs were observed among *CmC3H* members across the four groups (**Figure 3B**). As illustrated in **Figure 3B**, the protein motifs in the 10 *CmC3H* members of Group I were primarily motifs 1, 2, 3, and 10. The 9 *CmC3H* members of Group II mainly contained motifs 1, 4, 6, and 7. The 5 *CmC3H* members in Group III predominantly featured motifs 5 and 6. The 14 *CmC3H* members in Group IV primarily exhibited motifs 1 and 4. Moreover, according to the protein domain annotation results, each *CmC3H* family member contained at least one C3H domain (**Figure 3C**). In Group III, four *CmC3H* members contained five C3H domains situated at different positions within their protein sequences, and one member contained six C3H domains. For the other members, most of the C3H domains were distributed across one to three locations.

The gene structure of *CmC3H* family members is highly diverse, with the number of exons ranging from 1 to 16 and the number of introns ranging from 0 to 15 (**Figure 3D**). Significant differences were observed in the number of exons and introns among *CmC3H* members across the four groups, and also within members of the same group. In Group I, eight members had fewer than five exons, and two members had more than ten exons. In Group II, eight members had fewer than ten exons, and one member had more than ten exons. In Group III, one member had six exons, three members had seven exons, and one member had twelve exons. In Group IV, all 14 members had fewer than ten exons. Overall, the diversity in protein motifs and gene structures suggests that *CmC3H* family members may have diverse functions in regulating melon growth and development.

### 3.4 Synteny and homologous gene pair analysis of CmC3H family members

**Figure 4:**
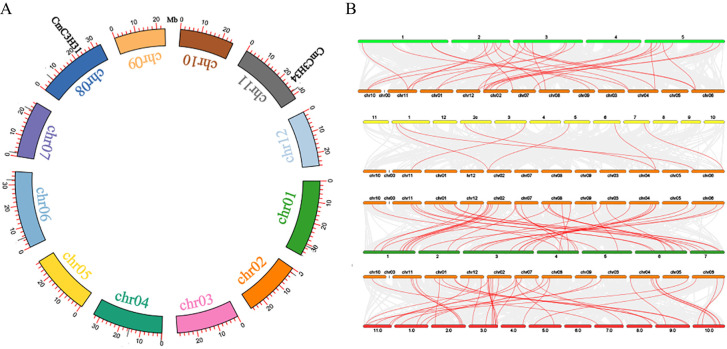
Synteny and homologous gene pair analysis of *CmC3H* family members. **(A)** Synteny circos plot of *CmC3H* family members within chromosomes. Red lines represent segmental duplication gene pairs. **(B)** Homologous gene pairs between melon and *A. thaliana*, rice, cucumber, and watermelon *C3H* family members. Red lines indicate homologous gene pairs.

A pair of segmental duplication genes was identified within the *CmC3H* family (*CmC3H31*/*CmC3H34*) (**Figure 4A**). Additionally, the *C3H* family members in melon were identified to have 20, 7, 39, and 38 homologous gene pairs with *A. thaliana*, rice, cucumber, and watermelon, respectively (**Figure 4B**; **Supplementary Table S3**). Furthermore, the Ka/Ks values for both segmental duplication gene pairs and homologous gene pairs were less than 1, except for 6 homologous gene pairs between melon and rice, for which Ka/Ks values were not calculable (**Supplementary Table S3**). These results indicate that *C3H* genes in dicotyledonous plants are likely to be highly conserved, whereas the conservation between monocots and dicots may be lower. Notably, the *C3H* homologous gene pairs between melon and cucumber, as well as between melon and watermelon, primarily exhibit a 1:1 distribution, indicating a high level of conservation of *C3H* family members across these three species during evolution.

### 3.5 Protein interaction and target gene prediction analysis of CmC3H family members

**Figure 5:**
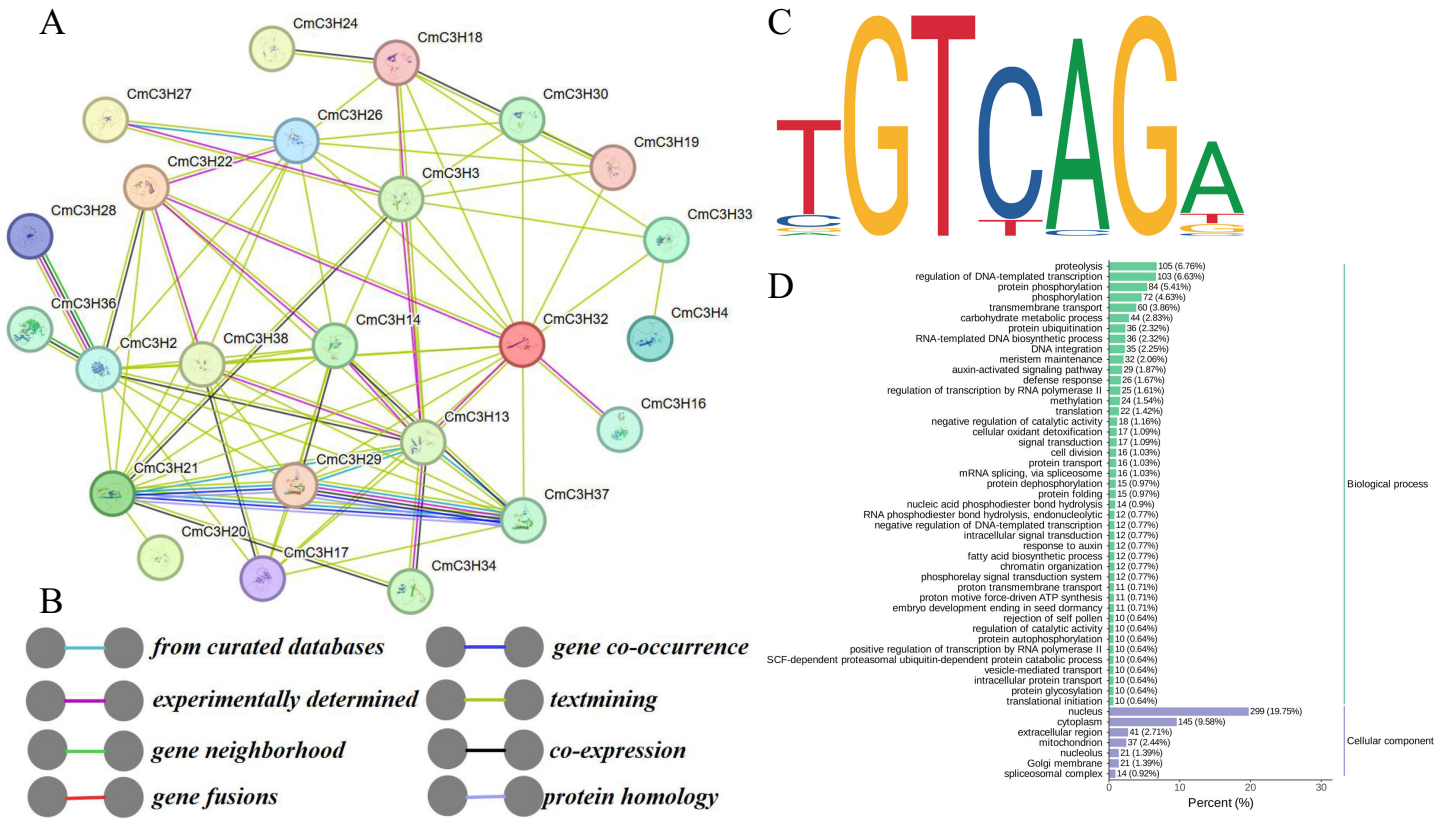
Protein interaction and target gene analysis of *CmC3H* family members. **(A)** Protein interaction network of CmC3H family members. **(B)** The meaning of different colored lines in the interaction network. **(C)** *GRF9* (*C3H*) transcription factor binding profile. **(D)** GO enrichment results for target genes.

Potential protein-protein interactions among CmC3H family members were predicted using the Arabidopsis database in the STRING tool (https://cn.string-db.org/) (**Figures 5A, B**; **Supplementary Table S6**). Potential interactions were identified among 29 CmC3H members, connected by 76 edges, forming a complex interaction network. For example, CmC3H26 potentially interacts with CmC3H27, CmC3H30, and other members. Conversely, some members, such as CmC3H28, show potential interaction with only a single member, CmC3H36. Notably, CmC3H13 was predicted to be a core gene, interacting with 13 other members. GO enrichment analysis revealed that CmC3H family members are involved in various protein functions, including nucleus (GO:0005634), ribonucleoprotein complex (GO:1990904), DNA binding (GO:0003677), metal ion binding (GO:0046872), nucleic acid binding (GO:0003676), and RNA processing (GO:0006396).

Using the binding motif profile of the Arabidopsis C3H12 transcription factor (MA1756.2), 3,243 target genes were identified in the melon genome, all of which matched the TGTCAGA sequence type (**Figure 5C**; **Supplementary Table S7**). GO enrichment analysis of these target genes (**Figure 5D**) indicated that, in the biological process category, most target genes were enriched in proteolysis (GO:0006508), regulation of DNA-templated transcription (GO:0006355), and protein phosphorylation. Five protein functional categories in the biological process category, including proteolysis (GO:0006508), regulation of DNA-templated transcription (GO:0006355), protein phosphorylation (GO:0006468), transmembrane transport (GO:0055085), and carbohydrate metabolic process (GO:0005975), were enriched with more than 40 target genes, based on the number of target genes enriched in Gene Ontology (GO) terms (**Supplementary Table S8**). In the cellular component category, the target genes were primarily enriched in the nucleus (GO:0005634), cytoplasm (GO:0005737), and membrane (GO:0016020). Overall, the potential interactions among CmC3H members suggest a coordinated regulatory network in melon, and the target gene analysis indicates that *CmC3H* genes may regulate a wide range of genes involved in various growth and developmental processes.

### 3.6 Cis-element analysis of CmC3H family members

**Figure 6:**
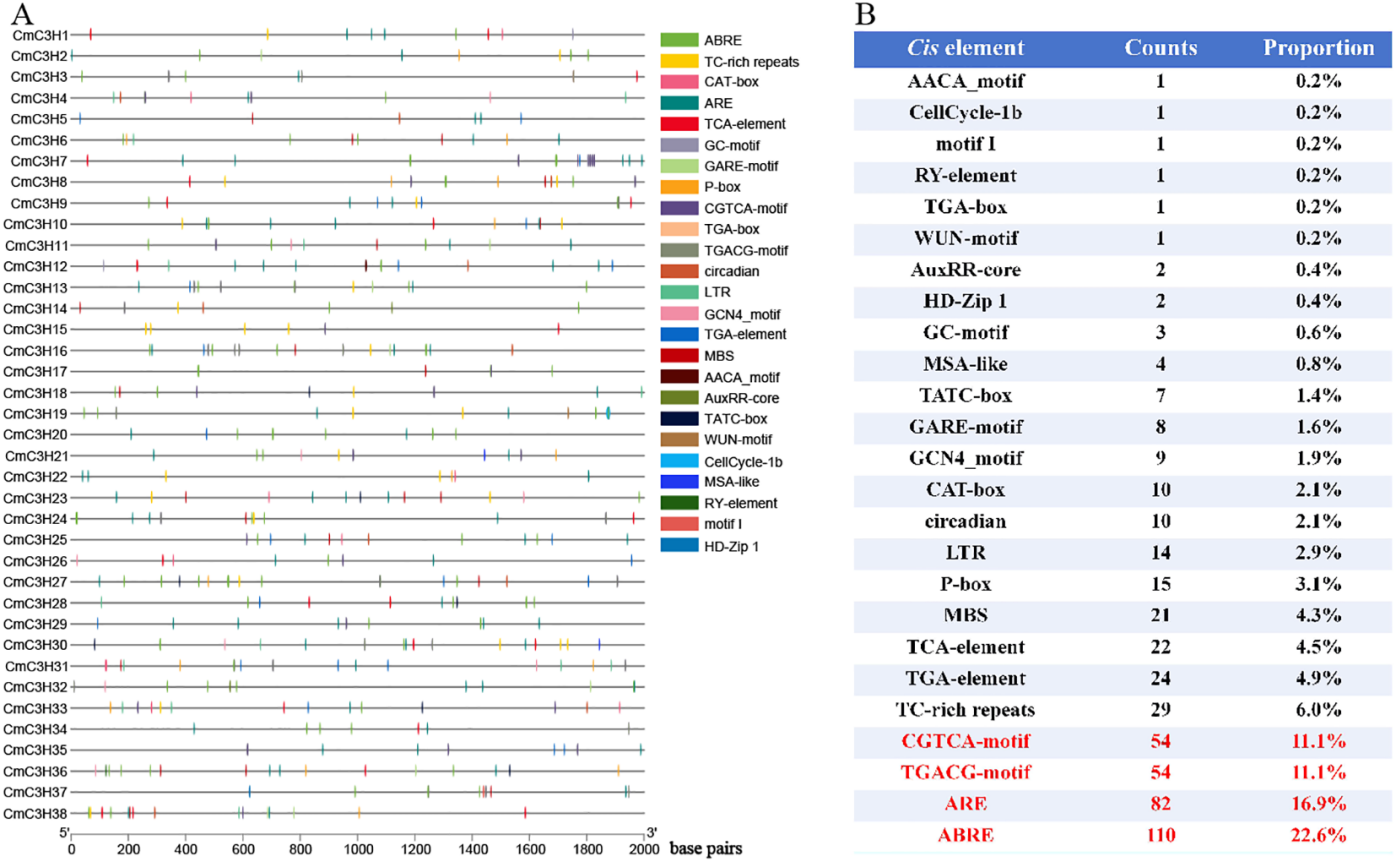
*Cis*-elements in *CmC3H* family members. **(A)** Distribution of hormone regulation, stress response, and growth and development-related *cis*-elements in *CmC3*H family members. **(B)** Quantitative distribution of hormone regulation, stress response, and growth and development-related *cis*-elements in *CmC3H* family members.

A total of 102 types of *cis*-elements were identified in the *CmC3H* family members. In addition to numerous light-responsive elements (such as G-box, MRE, and ATCT-motif), three major categories of *cis*-elements were classified: hormone regulation, stress response, and growth and development (**Figure 6A**; **Supplementary Table S4**). Hormone-regulatory *cis*-elements include ten types: TGA-element, TATC-box, TCA-element, ABRE, AuxRR-core, CGTCA-motif, TGACG-motif, GARE-motif, P-box, and TGA-box. Stress-responsive *cis*-elements include six types: TC-rich repeats, LTR, ARE, GC-motif, MBS, and WUN-motif. Growth and development-related *cis*-elements comprise nine types: MSA-like, circadian, RY-element, CAT-box, motif I, GCN4_motif, HD-Zip 1, AACA_motif, and CellCycle-1b.

Furthermore, the total number of hormone regulation, stress response, and growth and development-related *cis*-elements within the *CmC3H* family members was quantified. The CGTCA-motif (*cis*-element involved in MeJA-responsiveness), TGACG-motif (*cis*-element involved in MeJA-responsiveness), ARE (*cis*-element essential for anaerobic induction), and ABRE (*cis*-element involved in abscisic acid responsiveness) were particularly abundant, with 54, 54, 82, and 110 occurrences, respectively (**Figure 6B**). Overall, the presence of these *cis*-elements suggests that *CmC3H* family members may play a significant role in various physiological processes in melon, contributing to the normal growth and development of tissues under diverse conditions.

### 3.7 E>xpression pattern analysis of CmC3H family members

**Figure 7:**
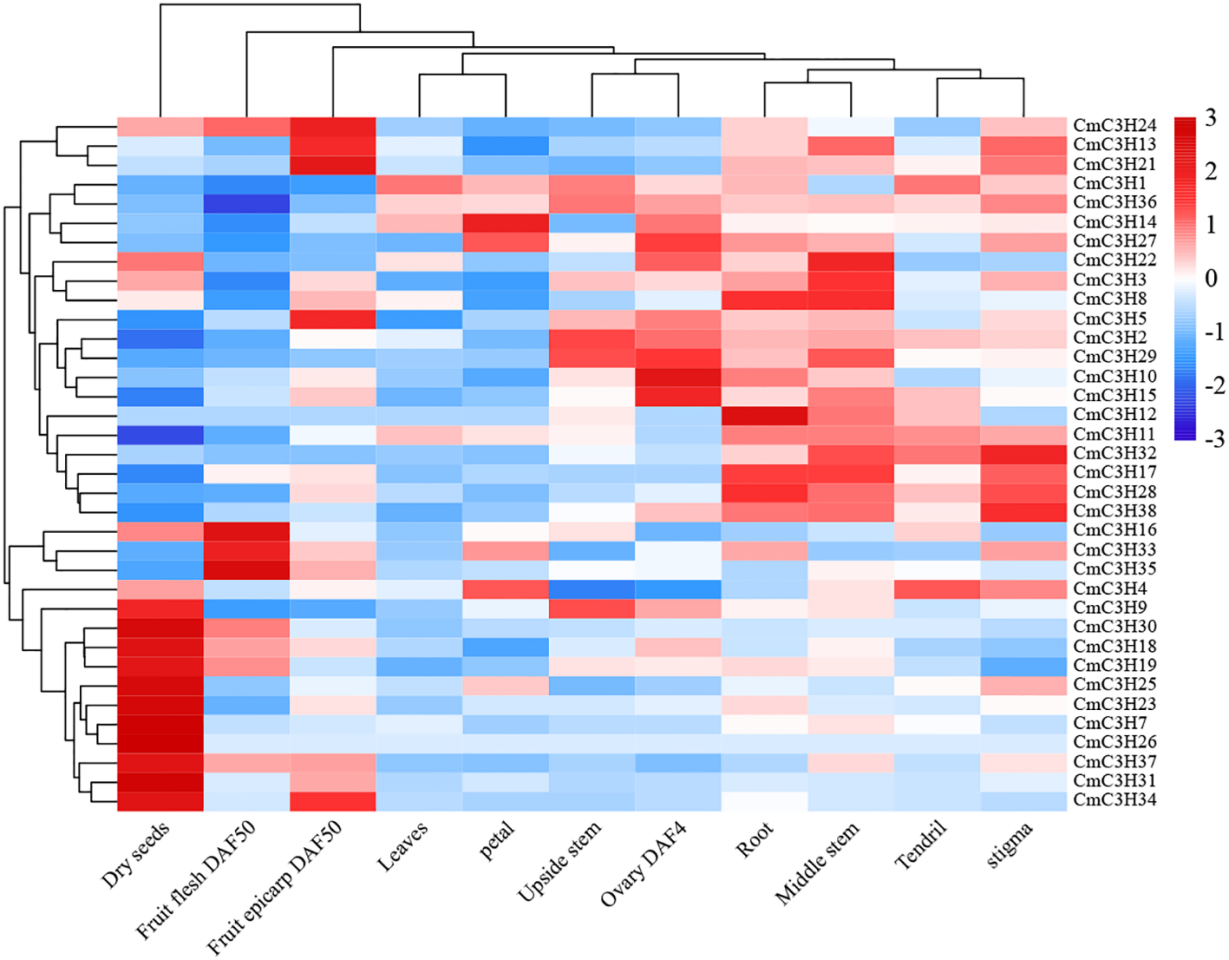
Heatmap of expression patterns of *CmC3H* family members in different tissues. Expression levels were normalized across rows, with color ranging from blue to red, representing low to high expression levels, respectively.

The FPKM values for 36 *CmC3H* family members were obtained across eleven melon tissues: dry seeds, root, middle stem, upside stem, leaves, tendril,petal, stigma, ovary at 4 Days After Flowering (DAF4), fruit flesh at 50 DaysAfter Flowering (DAF50), and fruit epicarp at 50 Days After Flowering (DAF50). A heatmap was generated to visualize their expression patterns (**Figure 7**; **Supplementary Table S5**). The results indicate that the expression levels of 13 genes (*CmC3H7*, *CmC3H9*, *CmC3H16*, *CmC3H18*, *CmC3H19*, *CmC3H22*, *CmC3H23*, *CmC3H25*, *CmC3H26*, *CmC3H30*, *CmC3H31*, *CmC3H34*, and *CmC3H37*) show significant expression in dry seeds. The expression levels of seven genes (*CmC3H8*, *CmC3H10*, *CmC3H11*, *CmC3H12*, *CmC3H17*, *CmC3H28*, and *CmC3H38*) are significantly expressed in root. The expression levels of twelve genes (*CmC3H3*, *CmC3H8*, *CmC3H11*, *CmC3H12*, *CmC3H13*, *CmC3H15*, *CmC3H17*, *CmC3H22*, *CmC3H28*, *CmC3H29*, *CmC3H32*, and *CmC3H38*) are significantly expressed in middle stem. The expression levels of five genes (*CmC3H1*, *CmC3H2*, *CmC3H9*, *CmC3H29*, and *CmC3H36*) are significantly expressed in upside stem.Only *CmC3H1* shows significant expression in leaves. The expression levels of three genes (*CmC3H1*, *CmC3H4*, and *CmC3H32*) are significantly expressed in tendril. The expression levels of three genes (*CmC3H4*, *CmC3H14*, and *CmC3H27*)are significantly expressed in petal. The expression levels of eight genes (*CmC3H4*, *CmC3H13*, *CmC3H17*, *CmC3H21*, *CmC3H28*, *CmC3H32*, *CmC3H36*, and *CmC3H38*) are significantly expressed in stigma. The expression levels of eight genes (*CmC3H2*, *CmC3H5*, *CmC3H10*, *CmC3H14*, *CmC3H15*, *CmC3H22*, *CmC3H27*, and *CmC3H29*) show significant expression in ovary at DAF4. The expression levels of five genes (*CmC3H16*, *CmC3H24*, *CmC3H30*, *CmC3H33*, and *CmC3H35*) are significantly expressed in fruit flesh at DAF50. The expression levels of five genes (*CmC3H5*, *CmC3H13*, *CmC3H21*, *CmC3H24*, and *CmC3H34*) are significantly expressed in fruit epicarp at DAF50. Notably, several genes, such as *CmC3H4*, *CmC3H11*, *CmC3H17*, and *CmC3H29*, are significantly expressed in multiple tissues. Overall, *CmC3H* family members exhibit distinct tissue-specific expression patterns in melon.

### 3.8 Quantitative expression analysis of CmC3H family members

**Figure 8:**
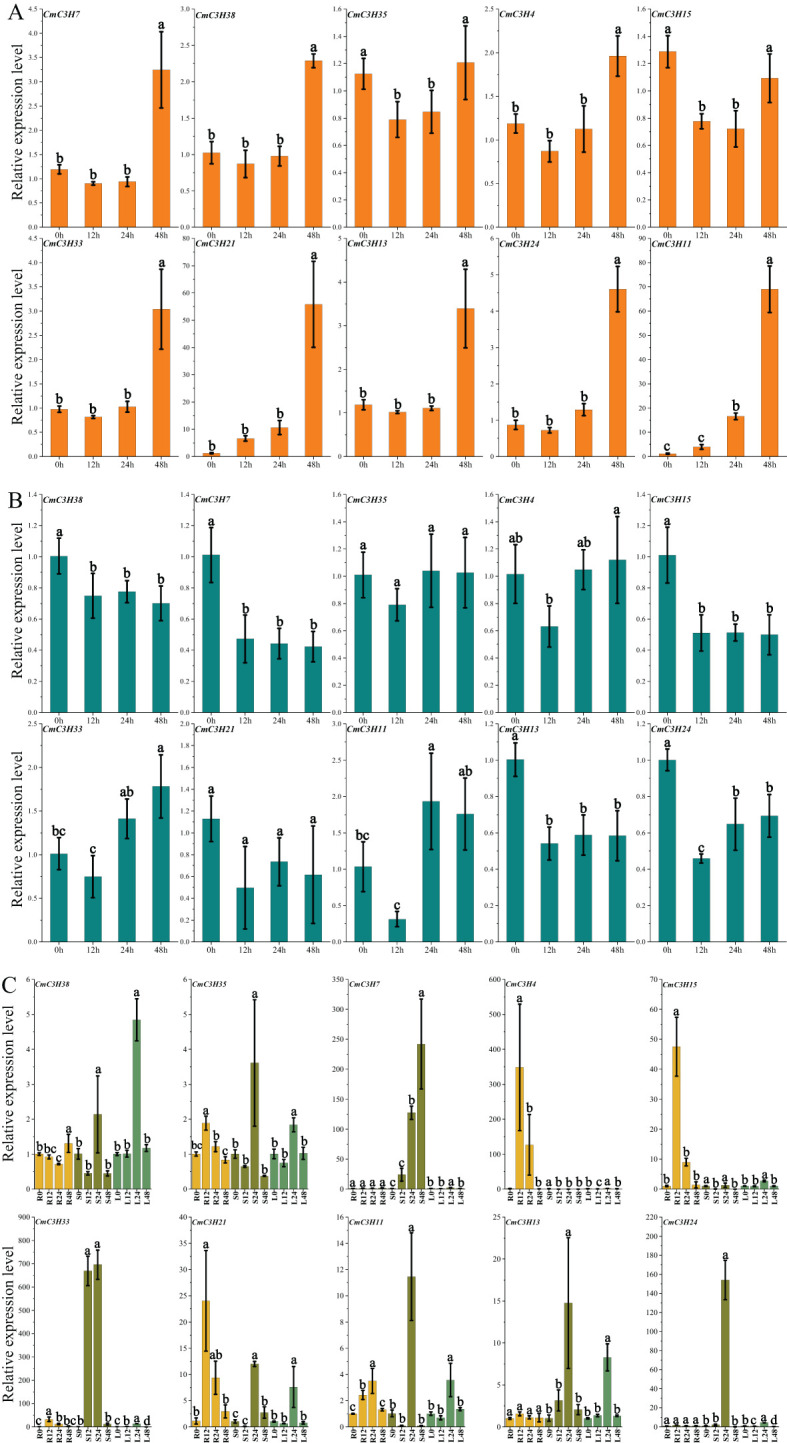
Quantitative Expression Levels of 10 *CmC3H* Genes at Four Time Points. **(A)** Expression levels under drought stress. **(B)** Expression levels under lead stress. **(C)** Expression levels in roots (R), stems (S), and leaves (L) following *Fusarium wilt* infection. Bars with lowercase letters above the columns indicate significant differences among the treatments at p < 0.05.

The expression levels of 10 *CmC3H* genes in melon leaves were measured over time under drought stress treatment (**Figure 8A**; **Supplementary Table S9**). The results showed that the expression levels of six genes (*CmC3H4*, *CmC3H7*, *CmC3H13*, *CmC3H24*, *CmC3H33*, and *CmC3H38*) exhibited significant changes at 48 hours, with expression levels ranging from 2 to 5 times those at 0 hours. Notably, the expression levels of *CmC3H11* and *CmC3H21* increased over time, particularly at 48 hours, where their expression levels were over 50 times higher than at 0 hours. In contrast, following lead stress treatment, the expression levels of the 10 *CmC3H* genes in melon leaves exhibited relatively minor changes over time, showing a general trend of decreasing expression, indicating that expression was suppressed (**Figure 8B**; **Supplementary Table S9**). Expression changes of the 10 *CmC3H* genes were further examined in the roots, stems, and leaves of melon under *Fusarium wilt* treatment (**Figure 8C**; **Supplementary Table S9**). The results showed that the expression levels of five genes (*CmC3H4*, *CmC3H15*, *CmC3H33*, *CmC3H21*, and *CmC3H11*) increased significantly in roots at 12 and 24 hours, particularly *CmC3H4*, whose expression increased by approximately 348 and 126 times at 12 and 24 hours, respectively, compared to 0 hours. In the stems, the expression levels of eight genes (*CmC3H7*, *CmC3H35*, *CmC3H38*, *CmC3H33*, *CmC3H21*, *CmC3H11*, *CmC3H13*, and *CmC3H24*) increased significantly at 24 hours, with *CmC3H24* and *CmC3H33* increasing approximately 669 and 153 times, respectively, compared to 0 hours. Additionally, *CmC3H33* expression at 48 hours increased approximately 696 times compared to 0 hours. In the leaves, the expression levels of nine genes(*CmC3H7*, *CmC3H35*, *CmC3H38*, *CmC3H15*, *CmC3H33*, *CmC3H21*, *CmC3H11*, *CmC3H13*, and *CmC3H24*) significantly increased at 24 hours following *Fusarium wilt* treatment.

### 3.9 Subcellular localization of CmC3H21

**Figure 9:**
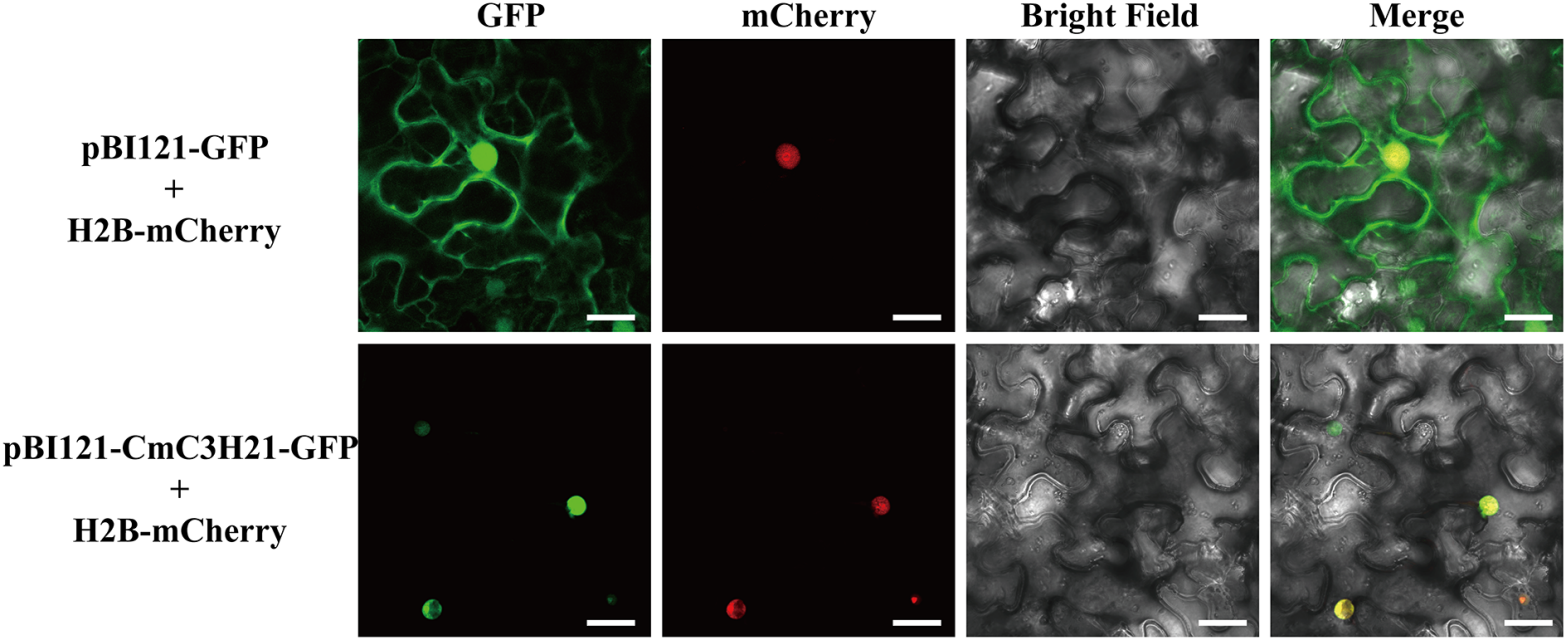
Subcellular localization of *CmC3H21*, with green (pBI121-CmC3H21-GFP) and red (nuclear marker H2B-mCherry) fluorescence signals (scale bar = 25 μm).

Based on the aforementioned results, we selected *CmC3H21* for further subcellular localization experiments. According to bioinformatics predictions, the CmC3H21 protein is likely localized in the nucleus (**Supplementary Table S1**). To validate this prediction, we employed the *Agrobacterium* infiltration method to introduce the protein fusion into tobacco leaves to observe the subcellular localization of *CmC3H21*. The results showed that CmC3H21-GFP was co-expressed with mCherry in the nucleus(**Figure 9**), indicating that *CmC3H21* is expressed in the nucleus.

## 4 Discussion

Zinc finger protein *C3H* genes are extensively involved in plant development, signaling, and responses to both biotic and abiotic stresses (Han et al., 2021). To date, numerous studies have investigated the *C3H* gene family in various plant species. However, research on the *C3H* gene family in melon remains underexplored. This study aims to address this gap by investigating the *C3H* gene family in melon. A total of 38 *CmC3H* genes were identified in melon, a number comparable to closely related species such as cucumber and watermelon, but significantly fewer than those in *A. thaliana* (68) (Wang et al., 2008), potato (50) (Deng et al., 2023), and *Pyrus betulaefolia* (17) (Liu et al., 2020). The *CmC3H* family members are unevenly distributed across the 12 chromosomes, with no significant correlation between chromosome length and the number of *CmC3H* genes. These findings are consistent with the distribution patterns of *C3H* genes observed in other species (Deng et al., 2023; Liu et al., 2020). Additionally, 17 *CmC3H* genes are located in regions characterized by low gene density on the chromosomes. No studies have examined the distribution of *C3H* family members across regions of varying gene density in other species. *CmC3H* members exhibit considerable variation in the number of amino acids, protein molecular weight, theoretical isoelectric point, instability index, and aliphatic index. Subcellular localization results predominantly indicate nuclear localization, consistent with findings for *C3H* family members in species like potato (Deng et al., 2023). The phylogenetic analysis did not reveal distinct classifications within the *C3H* gene family (Zhang et al., 2013; Liu et al., 2014). Consequently, the *CmC3H* members were classified into four groups (Group I, Group II, Group III, and Group IV) based on the phylogenetic tree of the *C3H* family in potato. High clustering conservation was observed among the *C3H* members from melon, cucumber, and watermelon within the same group. Additionally, significant sequence diversity exists among the *CmC3H* members within the same group. Gene function is closely linked to conserved motifs and gene structure within their protein sequences. Motif analysis of *CmC3H* family members revealed substantial diversity in gene sequences and significant differences in the number of exons and introns among the genes. These findings are consistent with previous reports on *C3H* family members in other species (Liu et al., 2020). In conclusion, the results suggest that *CmC3H* gene sequences have undergone significant differentiation during evolution, likely due to functional divergence driven by environmental adaptation.

Gene duplication is a major factor in the expansion and evolution of gene family members, contributing to the adaptation of species to environmental changes and the maintenance of normal life processes. 5, 15, 16, and 24 pairs of segmentally duplicated genes have been identified in the C3H family in four species: *Solanum tuberosum* (Deng et al., 2023), *Zea mays* (Peng et al., 2012), *Panicum virgatum* (Yuan et al., 2015), and *Vitis vinifera* (Wang et al., 2014). In the *CmC3H* family, only one pair of segmentally duplicated genes and no tandemly duplicated genes were identified, suggesting that gene duplication may not be the main factor driving the expansion of *CmC3H* family members. Melon shares 20, 7, 39, and 38 pairs of *C3H* homologous genes with *A*. *thaliana*, rice, cucumber, and watermelon, respectively, indicating a clear distinction between dicotyledonous and monocotyledonous plants. Furthermore, the *C3H* genes of closely related species exhibit greater conservation. The Ka/Ks ratio serves as an effective method for studying the evolutionary selection of duplicated genes. The Ka/Ks ratios for both segmentally duplicated and homologous gene pairs were found to be less than 1, indicating that these duplicated genes have mainly undergone purifying selection and maintain a high level of functional conservation (Song et al., 2024). Transcription factors often regulate various response processes through protein-protein interactions (Alves et al., 2014). It was predicted that 29 CmC3H members might have potential protein interactions, offering a reference for future exploration of *CmC3H* interactions. Transcription factors regulate gene expression by binding to specific cis-regulatory sequences within the promoters of target genes. Research on the target genes of *C3H* transcription factors in plants is limited. In this study, 3,243 target genes were identified in the melon genome based on the binding sites of the Arabidopsis *GRF9* (C3H) transcription factor. These target genes are enriched in diverse GO functions and may play roles in regulating various response processes in melon, laying a foundation for further research into the interactions between *CmC3H* genes and other genes.

*cis-*elements within promoter regions regulate gene expression. The results of *cis*-element analysis indicate that *CmC3H* family members are involved in various functions, including melon growth and development, stress responses, and hormone regulation. Investigating gene expression during tissue development and under adverse environmental conditions is crucial for understanding molecular developmental mechanisms (Page and Minocha, 2005). In various species such as *A. thaliana*, rice, potato, and banana (Mazumdar et al., 2017), members of the *C3H* family exhibit tissue-specific expression patterns in tissues such as leaves, floral organs, and fruits. Similarly, *CmC3H* family members in melon exhibit significant tissue-specific expression across eleven tissues, including dry seeds, root, middle stem, upside stem, leaves, tendril, petal, stigma, ovary at DAF4, fruit flesh at DAF50, and fruit epicarp at DAF50. This suggests that these genes may play roles in the growth, development, and functional regulation of different melon tissues. *C3H* genes are widely involved in plant responses to both biotic and abiotic stresses, as shown by significant findings. In pepper, the C3H-type genes *PEPTY4* and *PEPTY5* are induced by heat stress (Tang et al., 2023b). The overexpression of the *BoC3H* gene can enhance salt tolerance in transgenic cabbage (Jiang et al., 2017). Transgenic expression of *PeC3H74* in *Arabidopsis* significantly improves drought resistance (Chen et al., 2020a). *PvC3H72* enhances cold tolerance in transgenic switchgrass by regulating the expression of genes in the ICE1-CBF-COR complex and ABA signaling pathways (Xie et al., 2019). *GhZFP1* overexpression in tobacco significantly increases salt tolerance and disease resistance in transgenic plants (Guo et al., 2009). *DgC3H1* overexpression improves cold tolerance in chrysanthemum (Bai et al., 2021). Similarly, six genes (*CmC3H4*, *CmC3H7*, *CmC3H13*, *CmC3H24*, *CmC3H33*, and *CmC3H38*) exhibited significant upregulation in melon leaves 48 hours after drought stress treatment, with *CmC3H11* and *CmC3H21* showing particularly marked increases. Notably, under lead stress, the expression levels of these ten genes declined, suggesting that *CmC3H* genes may not enhance melon’s ability to respond to ionic stress. Interactions among the transcription factors *GHZFP1*, *GZIRD21A*, and *GZIPR5* can enhance fungal disease tolerance in cotton (Guo et al., 2009). Several *CmC3H* genes in melon roots, stems, and leaves showed significantly increased expression levels 24 hours after *Fusarium wilt* infection, with *CmC3H24* and *CmC3H33* displaying particularly high expression levels. Notably, *CmC3H11* and *CmC3H21* exhibited significant expression responses in roots, stems, and leaves under both drought and *Fusarium wilt* stress, indicating that these two genes may contribute to enhanced resistance to both biotic and abiotic stresses in melon. Quantitative PCR can be used to detect gene expression levels and predict their potential functions (Yang et al., 2022). Our results suggest that *CmC3H24* and *CmC3H33* are promising candidate genes for improving resistance to biotic stress, particularly *Fusarium wilt*, while *CmC3H11* and *CmC3H21* are key candidate genes for enhancing resistance to both biotic and abiotic stresses in melon. The expression levels of *CmC3H* genes were validated under both biotic and abiotic stress conditions using quantitative fluorescence analysis, which identified candidate genes that serve as a foundation for subsequent functional studies. Transgenic experiments and subcellular localization assays are crucial for the validation of gene functions. However, these experiments were not conducted in this study due to certain limitations. Future research will prioritize these experimental validations to thoroughly elucidate the function of the *CmC3H* genes.

## 5 Conclusion

In this study, 38 *CmC3H* genes were identified based on the melon DHL92 v4.0 genome, and various aspects of the *CmC3H* family members were analyzed, including protein physicochemical properties, phylogeny, gene structure, protein motifs, gene duplication, and expression patterns. Phylogenetic analysis categorized the *CmC3H* family members into four groups, with members within each group showing high conservation in gene structure and protein motifs. Gene duplication may not be significant in the expansion of *CmC3H* family members. Finally, quantitative fluorescence techniques were employed to examine the expression patterns of ten *CmC3H* genes in melon under drought stress, heavy metal lead stress, and *Fusarium wilt* infection at four different time points. This analysis revealed several key candidate genes that may play an important role in enhancing melon resistance to both biotic and abiotic stresses. The results of this study provide important insights for exploring the characteristics and functions of *CmC3H* family members.

## Data Availability

The original contributions presented in the study are included in the article/**Supplementary Material**. Further inquiries can be directed to the corresponding authors.
